# Evaluating the use of a novel low-cost measurement insole to characterise plantar foot strain during gait loading regimes

**DOI:** 10.3389/fbioe.2023.1187710

**Published:** 2023-08-17

**Authors:** Sarah R. Crossland, Heidi J. Siddle, Claire L. Brockett, Peter Culmer

**Affiliations:** ^1^ School of Mechanical Engineering, Institute of Functional Surfaces, University of Leeds, Leeds, United Kingdom; ^2^ School of Medicine, Leeds Institute of Rheumatic and Musculoskeletal Medicine, University of Leeds, Leeds, United Kingdom; ^3^ Deparment of Mechanical Engineering, INSIGNEO Institute for in Silico Medicine, University of Sheffield, Sheffield, United Kingdom; ^4^ School of Mechanical Engineering, Institute of Design, Robotics and Optimisation, University of Leeds, Leeds, United Kingdom

**Keywords:** diabetes, shear, strain, plantar, digital image correlation frontiers

## Abstract

**Introduction:** Under plantar loading regimes, it is accepted that both pressure and shear strain biomechanically contribute to formation and deterioration of diabetic foot ulceration (DFU). Plantar foot strain characteristics in the at-risk diabetic foot are little researched due to lack of measurement devices. Plantar pressure comparatively, is widely quantified and used in the characterisation of diabetic foot ulceration risk, with a range of clinically implemented pressure measurement devices on the market. With the development of novel strain quantification methods in its infancy, feasibility testing and validation of these measurement devices for use is required. Initial studies centre on normal walking speed, reflecting common activities of daily living, but evaluating response to differing gait loading regimes is needed to support the use of such technologies for potential clinical translation. This study evaluates the effects of speed and inclination on stance time, strain location and strain response using a low-cost novel strain measurement insole.

**Methods:** The STrain Analysis and Mapping of the Plantar Aspect (STAMPS) insole has been developed, and feasibility tested under self-selected normal walking speeds to characterise plantar foot strain, with testing beyond this limited regime required. A treadmill was implemented to standardise speed and inclination for a range of daily plantar loading conditions. A small cohort, comprising of five non-diabetic participants, were examined at slow (0.75 m/s), normal (1.25 m/s) and brisk (2 m/s) walking speeds and normal speed at inclination (10% gradient).

**Results:** Plantar strain active regions were seen to increase with increasing speed across all participants. With inclination, it was seen that strain active regions reduce in the hindfoot and show a tendency to forefoot with discretionary changes to strain seen. Stance time decreases with increasing speed, as expected, with reduced stance time with inclination.

**Discussion:** Comparison of the strain response and stance time should be considered when evaluating foot biomechanics in diabetic populations to assess strain time interval effects. This study supports the evaluation of the STAMPS insole to successfully track strain changes under differing plantar loading conditions and warrants further investigation of healthy and diabetic cohorts to assess the implications for use as a risk assessment tool for DFU.

## 1 Introduction

The global diabetic population has increased significantly in recent decades with growth predicted to continue (International Diabetes Federation, 2019). With this comes a rise in the associated development of diabetic foot disease. From this population it is expected up to 25% will develop diabetic foot ulceration (DFU) within their lifetime (Armstrong et al., 2017). The associated healing times and treatment pathway requirements for DFU lead to a labour and cost intensive process with over £900 million spent annually in the UK market alone (Kerr et al., 2019), which is neither beneficial to the patient or healthcare provider. Prophylactic intervention is fundamental to reducing DFU rates, but is often unsupported in clinical practice due in part to poor evidence base and cost to implement across the at-risk diabetic population (Heuch and Streak Gomersall, 2016; Kerr et al., 2019; Bus et al., 2020). The current evidence base for orthotic intervention is focused on pressure as a predictor of ulceration risk to inform offloading (Bus et al., 2020). This has centered the development of diabetic foot risk assessment tools to solely focus on pressure. While elevated and sustained plantar pressures in DFU are well researched, there is often discrepancy between ulcer location and the peak plantar pressure site (Lavery et al., 2003). Shear stress on the foot is thought in part to contribute to this deviation in expected location (Jones et al., 2022), but remains little understood and is not measured in risk assessment of the diabetic foot due to the poor availability of measurement tools. Strain, in the context of plantar assessment, can be considered as the resultant deformation from the combination of normal plantar pressure and shear stress. It is postulated that plantar tissue shear stress contributes to ulcer formation mechanics through subjecting the tissue to fatigue based failure subsurface (Yavuz, 2014; Yavuz et al., 2015; Yavuz et al., 2017). In this way, strain of the plantar surface skin can be used to assess both known contributors to ulcer formation, pressure and shear stress.

The complexities seen in the feet of people with diabetes leads to a requirement of bespoke treatment approaches. This in turn drives the development of objective risk assessment tools that allow quantifiable metrics of the at-risk diabetic foot and allow for earlier prophylactic interventions to reduce DFU formation risk and work towards preventing long term escalation of treatment costs (Bus et al., 2020). Current approaches to quantify shear stress at the plantar surface utilise a wide range of technologies including capacitive sensors and strain gauges (Kärki et al., 2009; Rajala and Lekkala, 2014), but have not established a clinically viable tool (Yavuz et al., 2007; Jones et al., 2022). In-shoe approaches to quantifying shear strain have gained renewed interest, with a range of studies reflecting the drive for responsive technologies. Development ranges from full foot coverage arrays utilising tri-axial sensors (Wang et al., 2020; Wang et al., 2022), to anatomically focused low coverage piezoelectric or single axis sensors (Takano et al., 2014; Amemiya et al., 2016). There is a current gulf in technology addressing both the pressure and shear stress components of plantar load.

Assessment of peak strain, *in lieu* of shear stresses is important in characterising the at-risk diabetic foot, with numerous studies highlighting the role of shear in ulcer formation (Yavuz et al., 2015; Yavuz et al., 2017; Jones et al., 2022). Peak pressure was long considered the key metric in assessment, with higher peak pressures associated with increased ulcer risk (Veves et al., 1992). More recent studies have shown that whilst pressure is important, it is not the sole predictor of ulcer formation location, with shear stresses playing a significant role (Ledoux et al., 2013; Yavuz et al., 2015; Yavuz et al., 2017). High peak shear stress, in relation to peak strain, is associated with tissue responses that lead to callus formation, a predeterming factor for DFU, showing signs of fatigue failure to the tissues with a warming response that reduces the resistance to tissue breakdown (Yavuz et al., 2015). Whilst pressure time integral is considered alongside peak plantar pressure and average pressure in assessing DFU risk (Keijsers et al., 2010; Waaijman and Bus, 2012; Bus and Waaijman, 2013), the contribution of shear strain time integral remains unclear. Currently, there are limited systems available to measure strain *in lieu* of shear forces and no current clinically utilised techniques for data collection.Yavuz et al. (2008) employed a custom built sensor platform to measure normal and tangential forces simultaneously of the unshod foot during stance phase to derive pressure and shear time integrals for a diabetic and non-diabetic cohort. This showed by an increase in both time integrals for the diabetic population and led to calls for further investigation of temporal strain responses.

The current pressure data capture techniques are divided into two distinct focuses of shod or unshod measures. Whilst unshod measures can give an understanding of intrinsic pressures due to anatomical variances and gait deviations, they do not reflect the activities of daily living where footwear is worn. However, in clinic these pressure devices, including pressure plates (Abdul Razak et al., 2012), offer a convenient method of data capture with which to inform orthoses design. Shod pressure data allows data to be collected during these activities of daily living to provide a representative understanding of the pressure events acting upon the diabetic foot. Technologies including as pedar^®^ [Novel GmbH, Munchen Germany] pressure measurement insoles are currently used in clinical and research settings to achieve shod pressure data collection. Recent trends include the emerging market of pressure reporting insoles offering real-time feedback to inform user behaviour and minimise DFU risk (Chatwin et al., 2021). For both of these methods, the cost, initial set-up, calibration requirements and training are prohibitive factors to their implementation in a clinical environment.

The shod environment also presents influential factors which may instigate the formation of ulceration due to pressure and shear events leading to mechanical tissue stress (Lord and Hosein, 2000). The interfaces between the foot, sock and shoe must be considered in this instance, alongside the pressure changes brought about by the footwear design and the influence on tissue stress (van Netten et al., 2018). To begin to understand the effect on differing loading regimes to the plantar aspect of the foot within the shod environment, controlled speed and inclination trials have been employed (Kernozek et al., 1996; Segal et al., 2004; Warren et al., 2004; Ho et al., 2010) using the pedar^®^ pressure measurement insole. This method allows for a benchmark to be provided, allowing reporting of patterns in pressure deviation with changing speeds that reflect activities of daily living.

Current clinical pressure measurements systems, such as pedar^®^ [Novel GmbH, Munchen Germany], provide the functionality to monitor pressure response changes under differing loading regimes in the feet of people with diabetes. Recognition of the need to assess the plantar aspect during functional gait is seen with use of technologies such as pedar^®^ and should form a basis for future monitoring method requirements.

The development of the STrain Analysis and Mapping of the Plantar Surface (STAMPS) insole by Jones et al. (2023) bridges these gaps in the literature by allowing for strain assessment as a surrogate for the components of plantar load during gait. Digital image correlation (DIC), computer vision tracking of changes to an applied stochastic speckle pattern (Michael et al., 2009), is used here to quantify the cumulative effects of plantar loading in the form of strain imparted on a plastically deformable insole during gait. The aforementioned clinical need for a loading regime responsive assessment method drove the methodology to analyse the STAMPS insole response (Jones et al., 2023). Currently STAMPS has been optimised for functionality and feasibility tested at self-selected normal walking speeds and without a gradient. This paper uses the STAMPS insole technique as a responsive tool to evaluate changes in strain characteristics aligned to changes in walking speed and inclination, including stance time, strain location and strain response. The aim of the study is to assess the response of the STAMPS insole, under these controlled gait conditions.

## 2 Materials and methods

### 2.1 Study protocol

To provide a consistent achieved walking speed and inclination across all studies a Nordictrack C200 Treadmill was implemented for use. Trials were selected to be conducted at 0.75 m/s, 1.25 m/s and 2 m/s speeds to reflect a slow, lower bound normal and brisk walking pace. These values align with conducted treadmill trials to monitor pressure variance with speed during gait using pedar ^®^ (Segal et al., 2004) and also reflect the range of speeds that might be adopted in typical activities of daily living. Inclination was set to a gradient of 10% reflecting a mid value condition selected by Ho et al. (2010). It was decided that for the purpose of this study, the inclination trial would deviate from Ho et al. (2010) and be conducted at the ‘normal’ 1.25 m/s speed, to reflect the expected general gait reported in a slower population (Segal et al., 2004), such as may be expected in the ageing diabetic population. Due to safety limitations, the treadmill belt restricts starting at the target speed and instead provides an acceleration to reach this speed. The treadmill acceleration profiles were collated using image analysis, recorded using a Nikon D5300 with AF-S Nikkor Lens (Nikon), to track belt speed changes under the three speed conditions (Padulo et al., 2014).

#### 2.1.1 Insole manufacture

**FIGURE 1:**
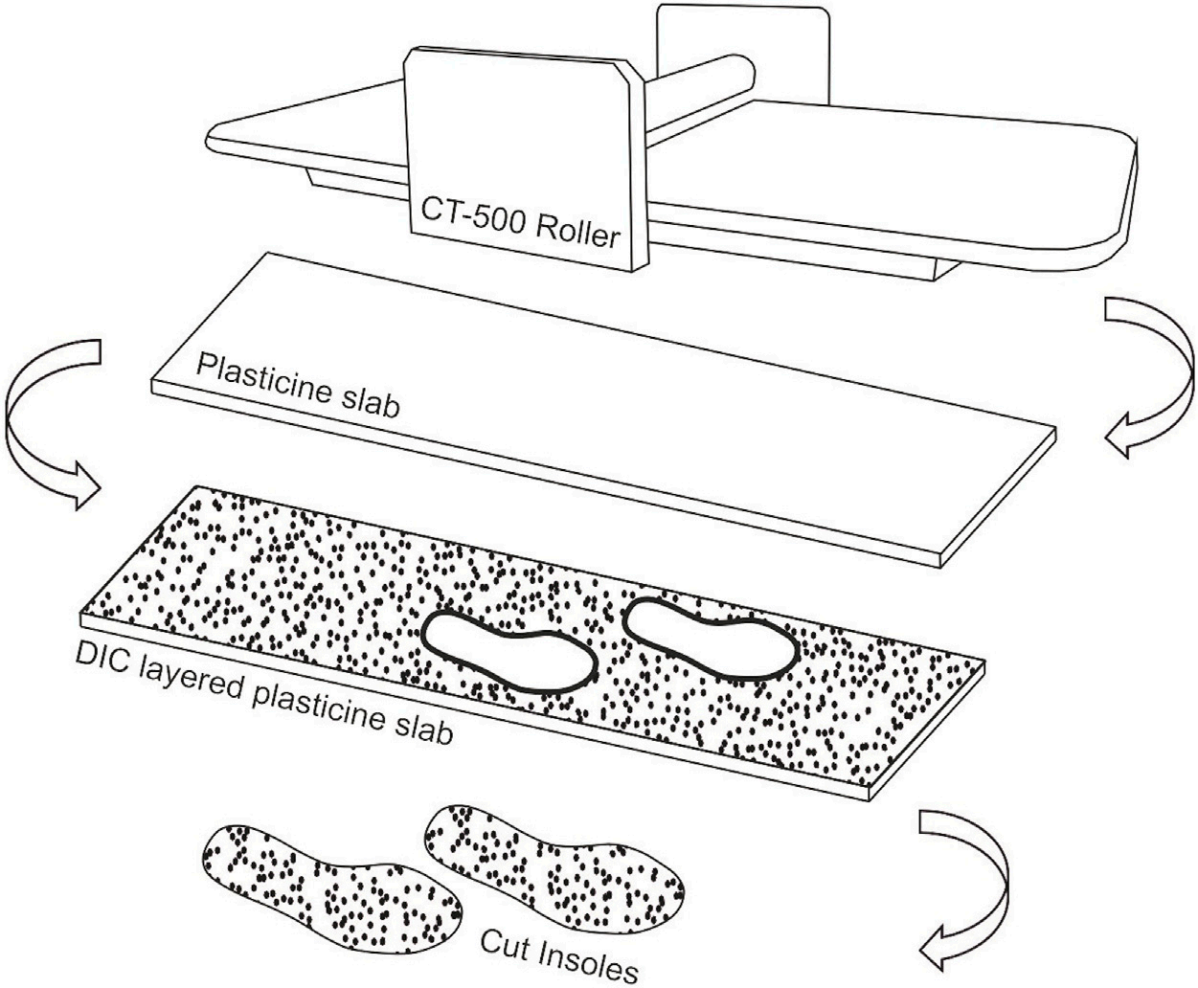
Insole manufacture process, showing schematic of stages from clay rolling slab, addition of DIC layer structure and cut out of insoles.

**FIGURE 2:**
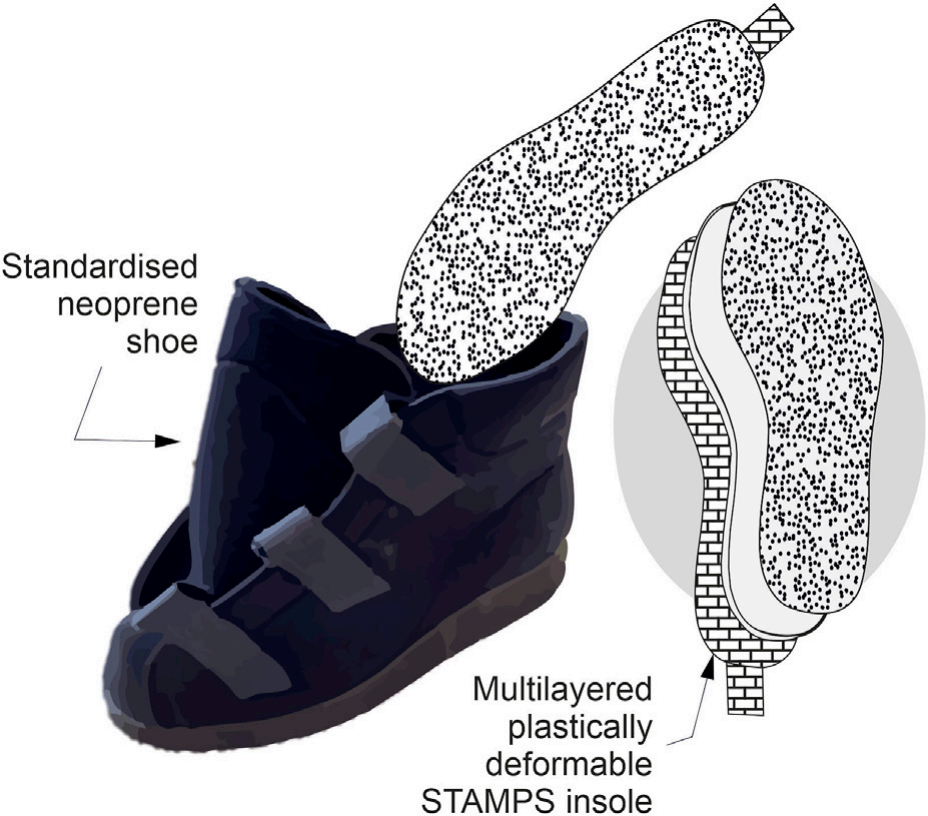
STAMPS insole layer view schematic showing standardised footwear utilised within the participant study.

The STAMPS plastically deformable insoles were prepared following the protocol described previously (Jones et al., 2023). A commercial clay roller (CT-500, North Star Polaris) was used to provide a targeted 5 mm thickness plasticine slab from which flat insoles were cut to size requirements Figure 1). Cross patterned Nylon mesh was used to reinforce the base of the insole and provide a posterior tab for ease of removal following use (Figure 2). The optimised computer generated stochastic speckle (Correlated Solutions Speckle Generator, v1.0.5), consisting of a 0.8 mm speckle with a 65% pattern density and 75% pattern variation, was applied via a thin film, 180 *μ*m, temporary tattoo (Silhoutte, United States) for the purpose of DIC. The insoles were allowed to rest for a period of 24 h minimum prior to use after moulding to allow for any temporal hardening effects (Chijiiwa et al., 1981). The insoles were then stored at a controlled 15 °temperature prior to use, in line with Jones et al. (2023) findings on storage and use optimisation for ten step gait studies.

#### 2.1.2 Participant study

**FIGURE 3:**
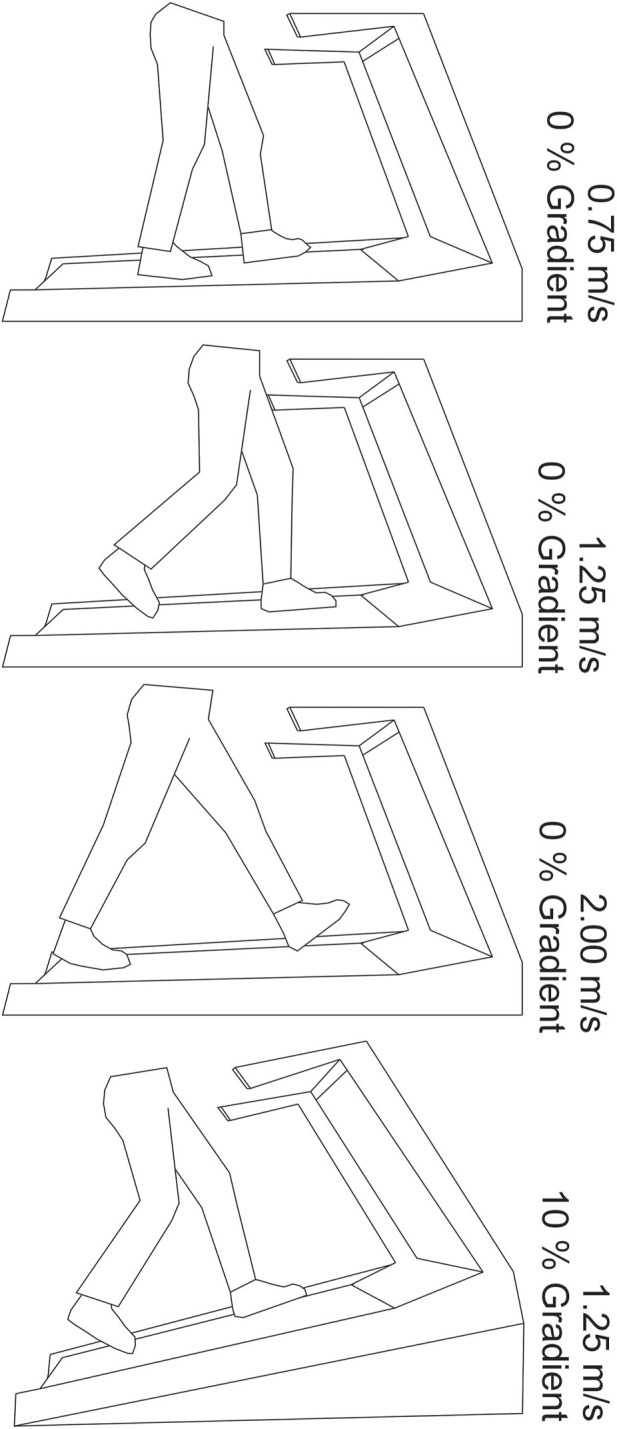
Depiction of speeds and inclination of treadmill during each phase of the study.

To verify the proposed study protocol, a participant cohort was recruited. The aim of which was to assess the ability of the STAMPS insole to effectively detect strain changes under differing loading regimes through controlled speed and inclination trials. A five participant non-diabetic cohort was recruited and provided consent, see Table 1. The University of Leeds Engineering and Physical Sciences joint Faculty Research Ethics Committee granted ethics approval (LTMECH-005) for the study design. The study assessed right foot stance phase loading solely, with each participant provided with a STAMPS insole for the right footwear with a contra-lateral sham insole in the left footwear to reduce inconsistency in leg length. Standardised neoprene footwear (Ninewells Boot, Chaneco LTD.) were used for consistency across all participants. Participants were asked to walk for ten steps on the right foot during each trial, inline with insole usability limits procured from insole optimisation (Jones et al., 2023). Three repeats were taken at each trialled speed and at inclination, with a new insole each trial due to the plastically deformable nature of the insoles rendering them single use. The target speed was achieved following an acceleration profile. With the lowest speed it enabled for a higher number of steps to be conducted at the target speed. This is compared to the highest speed where the time to reach full speed as increased and reflected in a lower number of steps at this speed. This disparity was between eight to five steps at target speed. Images were recorded of the STAMPS insole before and after undertaking each trial and participants were recorded using an camera recording at 50 fps (Nikon D5300, Nikon) to capture stance phase contact time. Figure 3.

**TABLE 1:** Participant characterisation data collated for speed and inclination treadmill study.

Participant	Gender	Height (m)	Weight (kg)	Age (Years [Months])	Shoe size (UK)
1	F	1.75	64.5	29 [3]	7
2	M	1.94	83.2	31 [11]	12
3	M	1.85	85.0	28 [6]	11
4	M	1.90	77.6	30 [7]	12
5	M	1.82	83.1	26 [9]	11

### 2.2 Plantar strain analysis

Commercially available DIC software (GOM Correlate 2019) was used for first stage post image DIC analysis to allow for the generation as insole strain maps. Strains were determined relative to the reference photo of the insole taken prior to each trial. For exportation of the data for post processing to derive positional strain values, an equidistant spread of points at 6.5 mm intervals was applied to each insole. The International Working Group on Diabetes recommends a sensor spread of 2 cm^2^ for pressure assessment of the diabetic foot specifically related to fixed sensor approaches, affording the STAMPS method increased resolution comparative this guideline (Bus et al., 2023).

**FIGURE 4:**
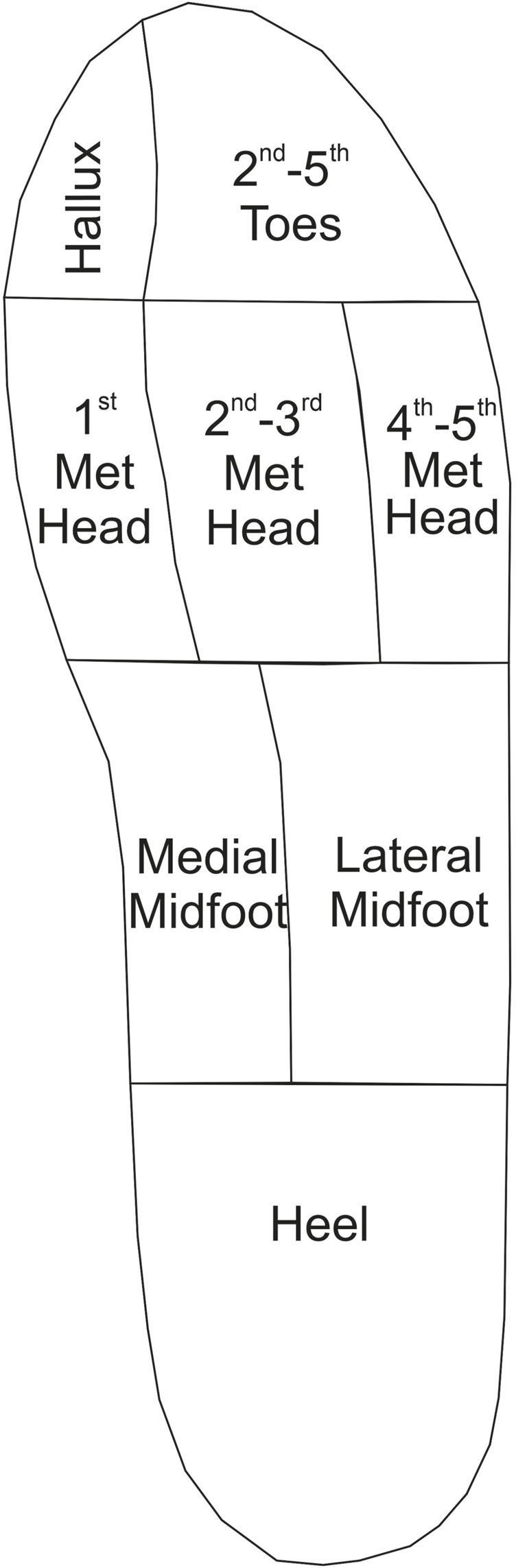
Anatomically defined regional mask which is used to catergorise strain output data for each participant trial.

Post processing was conducted in MATLAB (R2021b) for implementation of custom scripts to improve visualisation and allow for anatomical regional analysis of strain data. Pedar^®^ [Novel GmbH, Munchen Germany] used as a tool for risk assessing the diabetic foot, employs an Automask feature to divide the foot by regions of anatomically significance for segmented analysis in areas of DFU prevalence (?). Replicative masking across key anatomical landmarks was applied to the post-processed strain maps and aligned anatomical by a qualified orthotist (SRC). A reductive masking approach was then used to combine localised regions which would be difficult to distinguish clearly through assessment of the insole imaging. A resulting eight region mask was applied to determine strain outputs (Figure 4), covering: hallux, second to fifth toes, first metatarsal head, second and third metatarsal head, fourth and fifth metatarsal heads, lateral midfoot, medial midfoot and calcaneus. Average and peak strains across each segment were determined. The conducted MATLAB (R2021b) approach also allows for the recording of vector quiver plot for each trial, to provide information on the size and direction of the strain measured, though this is not presented within the scope of this study (Jones et al., 2023).

## 3 Results

**FIGURE 5:**
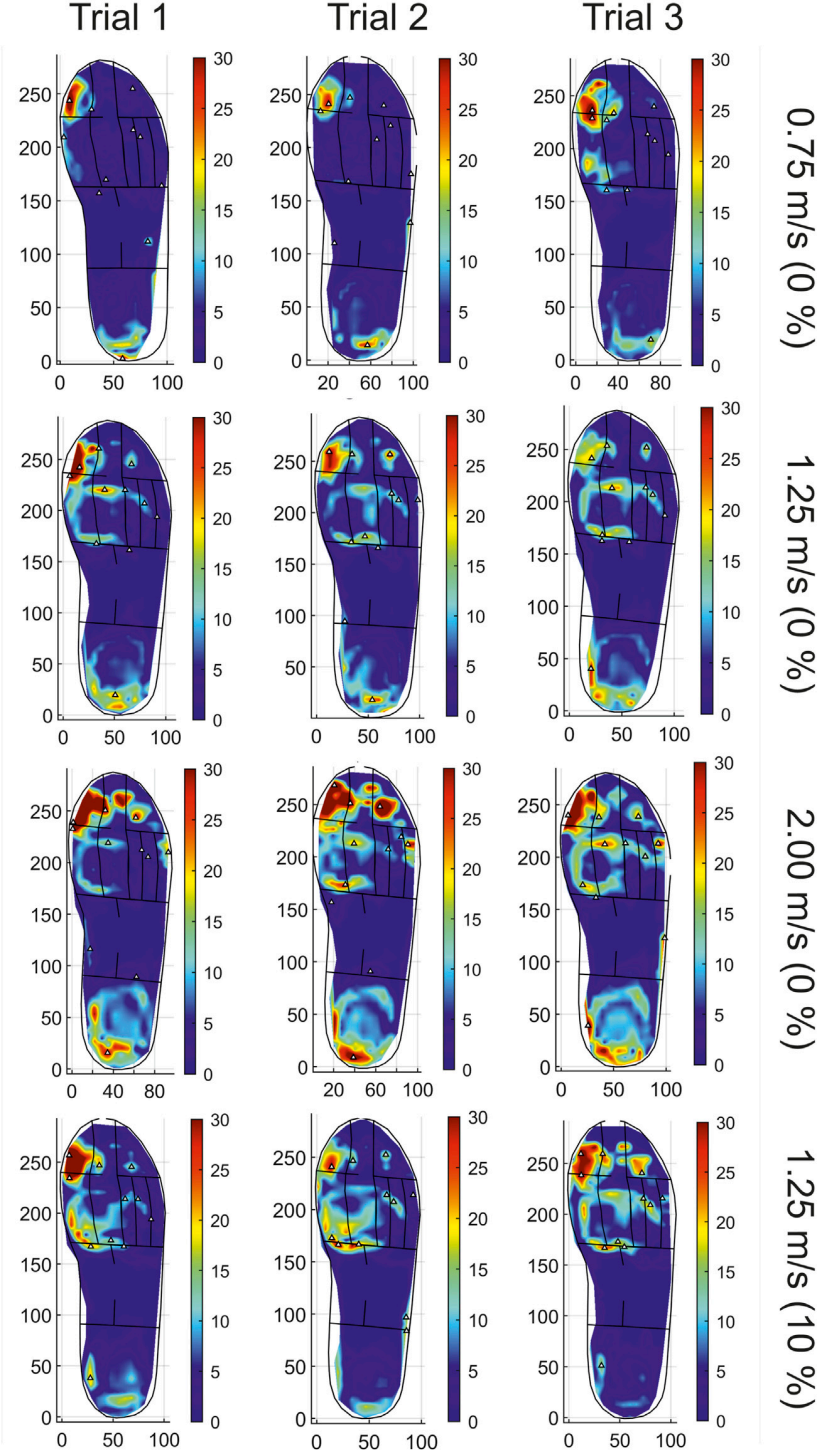
A representative example of strain profiles for repeated trialled speeds and inclinations for one participant (P03).

All trials were successfully completed for ten stance phases on the right foot by each participant. Figure 5 provides representative strain visualisation outputs from a single participant, showing the three repeated trials under each loading regime. The figure shows regions identified as being strain active increase with increasing speed, strain within the active regions also increases in line with the increasing speed. Figure 5 also highlights the variance between the two trials conducted at 1.25 m/s at 0% and 10% inclinations. Strain active regions are maintained in the forefoot with a reduction in activity seen in the hindfoot with increased inclination. These patterns are seen generally across all participants, with supplementary corresponding figures supplied for each of the remaining participants.

Tables 2, 3 show the averaged trial strains, standard deviations and percentage strain changes seen between speed changes (Table 2) and due to inclination change (Table 3). The trend between increasing speed and increasing average and peak strain can be seen for all participants in the majority of anatomical regions. There is some variance in the reported strain changes for inclination across differing anatomical regions and participants. All participants show a reduction in strain with increasing inclination at the rearfoot, in line with the reduction in strain active regions as seen in Figure 5.

**TABLE 2:** Anatomical regional average and peak strains to 2 d.p. and standard deviations (SD) averaged across all three repeat trials, with comparative percentage strain changes for all participants (P01-P05) at walking speeds of 0.75, 1.25 and 2.00 m/s.

		Average strain						Average strain - strain change (%)			Peak strain			Peak strain - strain change (%)		
		0.75 m/s	SD	1.25 m/s	SD	2.00 m/s	SD	[Slow to Normal]	[Normal to Brisk]	[Slow to Brisk]	0.75 m/s	1.25 m/s	2.00 m/s	[Slow to Normal]	[Normal to Brisk]	[Slow to Brisk]
P01	Hallux	6.13	3.65	8.79	5.17	15.46	8.60	43.35	75.78	151.99	11.57	19.73	32.11	70.57	62.72	177.55
	2nd-5th Toes	2.54	2.68	3.14	2.80	5.18	4.38	23.31	65.29	103.82	12.19	13.53	21.23	10.94	56.91	74.08
	1st Met Head	1.61	1.90	2.21	1.83	3.43	3.74	37.58	55.31	113.68	6.44	8.00	16.52	24.21	106.65	156.67
	2nd-3rd Met Heads	1.23	0.63	1.37	0.71	1.60	1.11	11.28	17.53	30.79	2.69	3.36	5.31	24.93	57.80	97.14
	4th-5th Met Heads	1.54	0.91	1.38	0.89	2.39	3.26	−10.35	73.82	55.84	4.01	3.89	14.23	−3.21	266.11	254.36
	Lateral Midfoot	0.77	0.67	0.68	1.00	0.90	0.75	−11.42	32.04	16.96	3.79	6.55	3.33	72.77	−49.12	−12.09
	Medial Midfoot	0.74	0.87	0.80	0.38	0.81	0.86	9.11	0.87	10.06	3.82	2.33	4.30	−39.09	84.78	12.55
	Heel	2.35	1.73	3.01	2.65	4.29	3.13	28.20	42.31	82.45	8.84	15.00	16.31	69.72	8.68	84.46
P02	Hallux	10.83	8.47	8.63	9.14	13.62	13.81	−20.32	57.81	25.74	35.11	51.32	51.01	18.92	46.20	73.86
	2nd-5th Toes	5.49	5.37	3.94	3.93	5.41	5.15	−28.28	37.41	−1.45	17.59	21.89	21.02	−40.85	24.48	−26.37
	1st Met Head	2.77	2.74	5.48	6.77	8.39	9.63	97.84	53.14	202.96	24.05	34.43	43.18	97.17	43.18	182.30
	2nd-3rd Met Heads	2.48	1.42	4.41	3.73	5.19	4.75	77.94	17.78	109.58	16.11	24.78	21.16	129.63	53.88	253.34
	4th-5th Met Heads	4.20	5.16	2.66	2.91	3.17	4.68	−36.58	19.06	−24.49	14.52	22.77	9.76	−37.54	56.83	−2.05
	Lateral Midfoot	1.86	1.83	1.23	1.22	0.85	1.00	−33.77	−30.76	−54.14	6.50	6.26	7.69	−19.75	−3.62	−22.66
	Medial Midfoot	0.57	0.61	0.69	0.96	0.59	0.44	21.31	−15.38	2.66	6.36	1.84	3.97	80.25	−71.09	−47.88
	Heel	3.28	2.96	4.17	4.09	5.40	4.63	27.15	29.62	64.81	24.16	24.53	14.92	63.78	1.56	66.34
P03	Hallux	12.10	11.15	14.17	10.79	24.92	18.94	17.12	75.86	105.97	38.23	31.73	63.35	−17.02	99.68	65.70
	2nd-5th Toes	2.83	3.15	4.04	4.49	8.07	9.82	42.68	99.65	184.87	15.29	25.08	42.45	64.04	69.30	177.73
	1st Met Head	6.36	5.52	8.23	6.36	10.11	6.98	29.47	22.79	58.97	25.71	30.04	32.76	16.83	9.06	27.41
	2nd-3rd Met Heads	2.63	1.78	6.47	5.38	6.17	4.88	146.27	−4.67	134.77	8.46	23.71	21.34	180.18	−10.00	152.18
	4th-5th Met Heads	1.86	1.26	3.45	3.03	5.53	6.57	85.29	60.51	197.40	5.66	15.46	29.21	173.11	88.95	416.05
	Lateral Midfoot	1.31	1.99	1.09	1.07	1.38	2.18	−16.82	27.13	5.74	15.88	4.60	12.73	−71.01	176.52	−19.84
	Medial Midfoot	1.38	1.70	1.34	1.70	1.55	1.73	−2.97	15.72	12.28	8.18	8.70	8.05	6.24	−7.37	−1.60
	Heel	5.47	4.92	7.16	5.65	10.11	7.21	30.78	41.26	84.744	27.12	28.60	37.99	5.46	32.82	40.08
P04	Hallux	12.12	17.53	23.78	22.03	19.11	22.38	96.26	−19.63	57.73	55.80	68.34	85.32	22.49	24.85	52.92
	2nd-5th Toes	7.68	11.74	9.03	10.82	14.39	13.55	17.61	59.31	87.35	59.28	51.71	62.91	−12.77	21.66	6.13
	1st Met Head	1.14	0.89	3.73	4.12	2.94	3.07	225.13	−21.17	156.28	4.32	17.10	11.94	295.99	−30.20	176.39
	2nd-3rd Met Heads	1.79	1.12	4.52	4.15	3.76	2.87	152.52	−16.77	110.16	4.64	17.55	13.83	278.20	−21.17	198.14
	4th-5th Met Heads	1.41	1.01	3.22	4.29	5.96	8.19	128.41	85.07	322.71	4.98	19.17	32.51	284.76	69.56	552.41
	Lateral Midfoot	0.41	0.27	0.52	0.78	0.41	0.68	25.41	−20.58	−0.40	1.53	4.80	4.91	214.87	2.15	221.65
	Medial Midfoot	0.76	0.92	0.47	0.56	0.41	0.28	−37.30	−13.76	−45.93	5.92	3.14	1.44	−46.89	−54.15	−75.65
	Heel	2.40	1.75	3.30	2.44	4.89	4.42	37.13	48.42	103.53	9.32	15.02	29.41	61.27	95.77	215.73
P05	Hallux	10.12	6.72	14.26	7.76	18.48	13.69	40.90	29.61	82.62	24.66	29.59	60.15	20.03	103.24	143.94
	2nd-5th Toes	2.98	3.68	3.70	4.20	11.06	11.75	24.24	198.66	271.07	20.77	19.79	58.84	−4.68	197.26	183.34
	1st Met Head	3.06	1.97	6.65	5.08	15.08	12.87	117.38	126.76	392.93	8.88	20.17	46.59	127.15	130.97	424.66
	2nd-3rd Met Heads	2.96	2.97	7.40	6.85	9.25	6.95	149.69	24.92	211.91	15.97	36.08	29.62	125.90	−17.88	85.50
	4th-5th Met Heads	4.16	6.70	7.31	9.43	5.78	8.11	75.93	−20.93	39.11	28.60	33.27	35.39	16.36	6.36	23.76
	Lateral Midfoot	1.86	1.30	1.82	1.30	1.83	1.46	−2.34	0.84	−1.52	5.31	4.91	6.44	−7.50	31.07	21.24
	Medial Midfoot	0.98	1.12	0.77	0.88	0.95	1.40	−21.25	23.22	−2.96	6.00	4.80	5.68	−20.07	18.38	−5.38
	Heel	3.67	2.99	3.93	3.34	7.73	5.91	6.83	96.87	110.33	20.18	21.31	35.45	5.61	66.37	75.70

**TABLE 3:** Anatomical regional average and peak strains to 2 d.p. and standard deviations (SD) averaged across all three repeat trials, with comparative percentage strain changes for all participants (P01-P05) at 1.25 m/s speed at 0% and 10% inclination.

		Average strain				Average strain - strain change (%)			Peak strain		Peak strain - strain change (%)		
		0%	SD	10%	SD	[0%–10%]	[0%–10%] Regional Average	Region	0%	10%	[0%–10%]	[0%–10%] Regional Average	Region
P01	Hallux	8.79	5.17	7.89	4.63	−10.26	−6.12	Forefoot	19.73	17.12	−13.24	17.44	Forefoot
	2nd-5th Toes	3.14	2.80	2.73	2.19	−12.84	—	—	13.53	10.09	−25.39	—	—
	1st Met Head	2.21	1.83	1.63	1.66	−26.09	—	—	8.00	9.05	13.22	—	—
	2nd-3rd Met Heads	1.37	0.71	1.39	0.88	2.12	—	—	3.36	4.49	33.43	—	—
	4th-5th Met Heads	1.38	0.89	1.60	1.36	16.48	—	—	3.89	6.96	79.17	—	—
	Lateral Midfoot	0.68	1.00	0.54	0.34	−21.25	−6.95	Midfoot	6.55	1.62	−75.20	24.99	Midfoot
	Medial Midfoot	0.80	0.38	0.86	0.87	7.35	—	—	2.33	5.25	125.19	—	—
	Heel	3.01	2.65	2.57	2.04	−14.74	−14.74	Rearfoot	15.00	10.44	−30.38	−30.38	Rearfoot
P02	Hallux	8.63	9.14	14.30	14.00	65.64	27.32	Forefoot	35.11	51.01	45.32	28.61	Forefoot
	2nd-5th Toes	3.94	3.93	3.97	3.66	0.88	—	—	17.59	21.02	19.55	—	—
	1st Met Head	5.48	6.77	8.14	10.61	48.60	—	—	24.05	43.18	79.55	—	—
	2nd-3rd Met Heads	4.41	3.73	5.68	4.31	28.80	—	—	16.11	21.16	31.38	—	—
	4th-5th Met Heads	2.66	2.91	2.47	1.92	−7.33	—	—	14.52	9.76	−32.77	—	—
	Lateral Midfoot	1.23	1.22	1.26	1.43	2.59	−0.52	Midfoot	6.50	7.69	18.32	−9.61	Midfoot
	Medial Midfoot	0.69	0.96	0.67	0.61	−3.63	—	—	6.36	3.97	−37.54	—	—
	Heel	4.17	4.09	3.48	3.00	−16.42	−16.42	Rearfoot	24.16	14.92	−38.24	−38.24	Rearfoot
P03	Hallux	14.17	10.79	16.15	14.00	13.95	18.68	Forefoot	31.73	44.14	39.14	9.75	Forefoot
	2nd-5th Toes	4.04	4.49	4.88	6.21	20.76	—	—	25.08	23.94	−4.53	—	—
	1st Met Head	8.23	6.36	10.83	7.73	31.60	—	—	30.04	32.25	7.36	—	—
	2nd-3rd Met Heads	6.47	5.38	6.83	4.99	5.53	—	—	23.71	20.98	−11.54	—	—
	4th-5th Met Heads	3.45	3.03	4.19	3.99	21.54	—	—	15.46	18.29	18.30	—	—
	Lateral Midfoot	1.09	1.07	1.46	2.44	34.42	86.05	Midfoot	4.60	14.40	212.70	236.90	Midfoot
	Medial Midfoot	1.34	1.70	3.19	6.57	137.67	—	—	8.70	31.40	261.10	—	—
	Heel	7.16	5.65	4.52	4.07	−36.81	−36.81	Rearfoot	28.60	20.16	−29.50	−29.50	Rearfoot
P04	Hallux	23.78	22.03	12.08	16.56	−49.21	−10.05	Forefoot	68.34	60.90	−10.89	−19.83	Forefoot
	2nd-5th Toes	9.03	10.82	10.35	11.00	14.59	—	—	51.71	60.21	16.44	—	—
	1st Met Head	3.73	4.12	2.61	2.30	−29.99	—	—	17.10	9.25	−45.91	—	—
	2nd-3rd Met Heads	4.52	4.15	4.79	3.46	5.88	—	—	17.55	14.09	−19.72	—	—
	4th-5th Met Heads	3.22	4.29	3.49	2.65	8.46	—	—	19.17	11.69	−39.05	—	—
	Lateral Midfoot	0.52	0.78	0.57	0.72	10.57	25.18	Midfoot	4.80	5.45	13.36	35.54	Midfoot
	Medial Midfoot	0.47	0.56	0.66	0.90	39.78			3.14	4.96	57.72		
	Heel	3.30	2.44	2.48	1.91	−24.80	−24.80	Rearfoot	15.02	10.13	−32.58	−32.58	Rearfoot
P05	Hallux	14.26	7.76	12.12	6.89	−14.99	−5.80	Forefoot	29.59	26.44	−10.67	2.86	Forefoot
	2nd-5th Toes	3.70	4.20	4.71	4.85	27.24	—	—	19.79	25.31	27.86	—	—
	1st Met Head	6.65	5.08	8.76	7.81	31.73	—	—	20.17	29.18	44.64	—	—
	2nd-3rd Met Heads	7.40	6.85	4.05	3.13	−45.23	—	—	36.08	14.28	−60.42	—	—
	4th-5th Met Heads	7.31	9.43	5.29	8.36	−27.73	—	—	33.27	37.57	12.91	—	—
	Lateral Midfoot	1.82	1.30	1.17	1.45	−35.83	−13.52	Midfoot	4.91	9.28	88.87	58.05	Midfoot
	Medial Midfoot	0.77	0.88	0.84	0.96	8.79	—	—	4.80	6.10	27.24	—	—
	Heel	3.93	3.34	2.92	2.65	−25.70	−25.70	Rearfoot	21.31	19.08	−10.48	−10.48	Rearfoot

**FIGURE 6:**
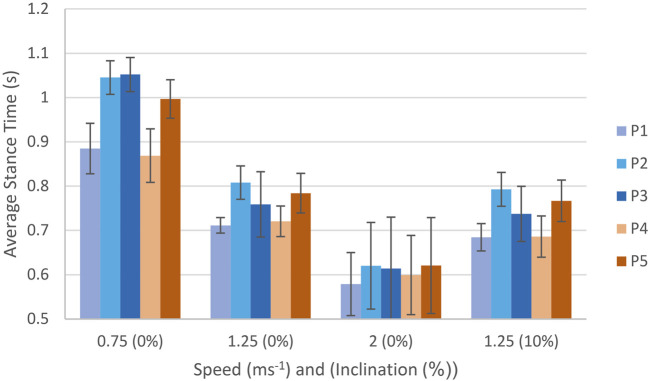
Average stance time per participant across all plantar loading regimes with associated standard deviations.

Average stance time, across all ten stance phases and over three repeated trials per loading regime (Figure 6). Decreases with increasing speed for all participants. A marginal decrease in average stance time is seen for all participants comparative between 1.25 m/s 0% to 1.25 m/s 10% inclination.

## 4 Discussion

The aim of the study was to utilise the STAMPS novel measurement insole to evaluate strain characteristic changes (Jones et al., 2023), including stance time, strain location and strain change, instigated through changes in walking speed and inclination. This was done specifically with the aim to assess the response of the STAMPS insole under controlled gait conditions (Jones et al., 2023). Strain is measured as a surrogate of shear stress. The strain responses captured using the STAMPS insole were compared to the capabilities of current pressure measurement systems used in DFU assessment, namely, the pedar^®^ [Novel GmbH, Munchen Germany] pressure capture insole. Studies by Segal et al. (2004) and Ho et al. (2010) using the pedar^®^ insole showed increased pressure with increasing speed. Strain captured by the STAMPS insole is related to plantar loading comprised of pressure and shear strain contributions, it is therefore expected that with an increasing speed and associated pressure, an increase in strain would be observed.

Strain outputs for all participants, Table 2, confirm this expectation by showing increased strain consistently across all trials with increasing speed (Segal et al., 2004). Average stance time, Figure 6, reduces with increasing speed which is concurrent with expectations for normal gait (Kirtley et al., 1985; Olney et al., 1994; Roth et al., 1997; Demur and Demura, 2010). Inclination strain change reductions across participants for both average and peak strains, Table 3, align with the reduction in rearfoot strain active locations seen, example shown in Figure 5. Though there is variation in participant strain changes to the mid and forefoot in both average and peak strain, the peak strain changes in these regions tend to show a general increase for participants at 10% gradient. Ho et al. (2010) reported the effect of inclination on pressure showed reduction in peak pressures at the rearfoot at inclinations of 5%, 10% and 15% gradient, with changes to the location of strain actives regions present from the 0% gradient. This is congruent with the strain changes reported from this study. The comparative literature is limited to studies of in-shoe pressure utilising a treadmill approach and pedar insole, other approaches to pressure measurement may differ in their reported values. Whilst the use of pedar reflects the current gold standard approach for clinical assessment of in-shoe pressure, it must be noted that pressure measurement field is a wide market of existing and emerging technologies and as such, differing reported outcomes. Ankle dorsiflexion increases with increasing inclination during stance, with decreased braking forces and increased late stance phase propulsive forces also seen (Tulchin et al., 2010). In turn, vertical and anteroposterior shear ground reaction forces (GRFs) increase with inclination (McIntosh et al., 2006). These changes reflect the reduced strain seen at the rearfoot and increased strain at the forefoot during this study.

The gradient chosen for this study does not necessarily reflect the daily gait activities of a person at-risk of diabetic foot complications. A 10% gradient can be considered a moderate-large incline, and with high-risk diabetics skewing towards and older and less mobile population, their daily activity locations may be centred around lower gradient terrains such as the home. Further studies at a reduced gradient and during other activities such as stair ascending and descending would be beneficial to understand strain response variations.

The regional strain percentage decreases reported for some participants across the trialled conditions for speed and gradient do not necessarily reflect the strain changes across the foot as a whole. While a tendency for the whole foot to show increasing strain with increasing speed is seen, the decreasing regional values may reflect the loading changes required of the foot to offset gait deviations undertaken in response to increasing speed as seen in Kernozek et al. (1996) shod pressure study. Likewise the same can be seen in the response of the foot with inclination reported by Ho et al. (2010).

Peak plantar pressure has been considered in DFU analysis alongside pressure time integral as metric used to determine DFU risk (Keijsers et al., 2010; Waaijman and Bus, 2012; Bus and Waaijman, 2013). Peak pressure is a poor predictor of DFUs when considered independently, with peak shear stress contributing to ulcer risk through fatigue failure to the tissues, through localised heating and reduced resistance to breakdown, with increased callus formation (Yavuz et al., 2015). Utilising peak strain, the resultant deformation from plantar pressure and shear stress, allows for the contribution of both these components to be considered in the tissue mechanics leading to DFU risk.

Whilst the STAMPS insole does not allow for the recording of strain changes during stance phase for direct calculation of strain time integral, and instead provides a reflection of cumulative strain, analysis of regional average strain in relation to longevity of stance can be considered *in lieu* of this metric. The small cohort in this study does not allow for statistical analysis and reporting of significance, but can be considered a benchmark study to assess this metric in a larger healthy cohort.

A limitation of this study is the use of a treadmill to standardise the walking speeds achieved, however, participants adopted all chosen pace settings comfortably. Whilst it achieves that aim, it can result in altered gait biomechanics compared to non-treadmill walking to compensate for controlled speed and belt movement (Lee and Hidler, 2008). Therefore the strain profiles may be altered in comparison to strain results recorded due to natural speed changes. A study analysing the strain response of self-selected slow and faster walking speeds should be run to address this.

The acceleration profile of the belt was dependent on the target outcome speed, with lower speeds having a lower initial rate of acceleration comparative to the higher target speed. The acceleration profiles were also non-liner in presentation. Speeds in all three targeted trials were reached prior to half of the steps being completed in all cases, with the plastically deformable STAMPS insole recording peak strains occurring at the target speed. The acceleration profile of the treadmill, rather than immediate target speed reached, does enable the full ten steps undertaken in the trial to be conducted at the target speed. The increased time it takes to reach higher speeds, due to the same initial starting speed, means that a differing number of steps are completed at the target velocity in relation to which speed the trial was conducted at. The result this has on the strain outcomes should be minimal due the plastic deformation of the insole, however this cannot be measured in the remit of this study. Acceleration profiles can be present when achieving self-selected walking speeds within brisk activities of daily living. These acceleration profiles are often not seen with slower self-selected walking speeds, which can be achieved instantaneously from initiation of gait. These natural deviations from a single continuous walking speed, whilst not directly represented in the study due to the controlled acceleration profile, should be considered when assessing the feasibility of strain data capture methods to respond to change in speed during gait events.

Inclined walking on a treadmill leads to biomechanical gait deviations compared to walking on a ramp. These changes include shorter steps and shorter stance times, alongside increased hip and knee flexion angles. No significant changes are seen to ground reaction forces, leading to the assumption of consistency in plantar strain between walking on both inclined surfaces. However, the gait deviations show an unnatural gait pattern which may be reflected in differing active plantar strain regions. Whilst treadmill walking helps to maintain set speeds to compare between participants, natural ramp walking should be trialled in future to address this (Strutzenberger et al., 2022).

The participant study was conducted with a small cohort of healthy participants to assess both if there was a difference seen in strain response and stance time, and if this was measurable using the STAMPS insole technique. Due to this limited study, no statistical significance can be attributed to the strain differences reported, a larger cohort study is required with appropriate power to further this work. Therefore, at this stage no definitive conclusions can be drawn relating to shear loading during normative gait. To work towards translation of the data to reflect a range of activities of daily living in clinical decision making process, a range of inclinations should be trialled over a larger population.

A singular inclination value is studied at a relatively steep incline of 10%, to report how inclination affects strain response. Beyond this the cohort demographic only covers a young adult, non-diabetic population, meaning that it is not generalizable to other cohorts. However the opportunity to measure strain and potentially reduce the incidence of DFU requires further studies in this population. Development of 3D DIC image capture is also required to enhance the analysis of the insole deformation profiles, ensuring the recorded strains reflect a true representation of regionalised strain response. This is particularly important in relation to potential future clinical translation to allow for strain data to support DFU risk assessment and treatment pathways.

The standard deviations presented when assessing the segmented anatomical regions are relatively large. This reflects the spread of strain within these regions and the specificity of skin response to gait. This shows that anatomical regions, whilst a helpful indicator of key regions on concerns, may overgeneralise the strain. The use of strain maps alongside regional masking of strain data can alleviate this, to allow for more specified targeting of locations of interest if required.

Prior use of the insole has been limited to normal self-selected walking speeds to reflect the average patients gait speed undergoing activities of daily living, but expanding this to reflect the altered activities of daily living experienced by diabetic cohorts due to foot structure, deformities and gait deviations is important. Assessment of the characteristics of these daily activities has been increasing in recent years and emphasises the importance of characterising gait beyond a research setting (Rozema et al., 1996). Focus should also be given in future research to assess anatomical region displacement within the segmented mask under speed variation, to reflect movement of the foot in relation to the insole. A larger scale participant study is required to support this approach. Understanding strain response to speed and inclination offers the opportunity to provide informed treatment approaches, such as footwear design, to optimise plantar loading for reduced DFU risk. With the increase in biomechanical assessments of activities of daily living, the clinical translation potential of the STAMPS insole could also be optimised to explore pathology and disease progression through plantar loading.

## Data Availability

The data is available in a data respoistory https://doi.org/10.5518/1309.
