# Supplementary material for: Evaluating the use of a novel low-cost measurement insole to characterise plantar foot strain during gait loading regimes
Source: Front Bioeng Biotechnol. 2023 Aug 17;11:1187710. doi: 10.3389/fbioe.2023.1187710 (PMC10469908; doi:10.3389/fbioe.2023.1187710)
Supplement: Supplementary file 1 [file DataSheet1.PDF]

Supplementary Material

1 SUPPLEMENTARY FIGURES

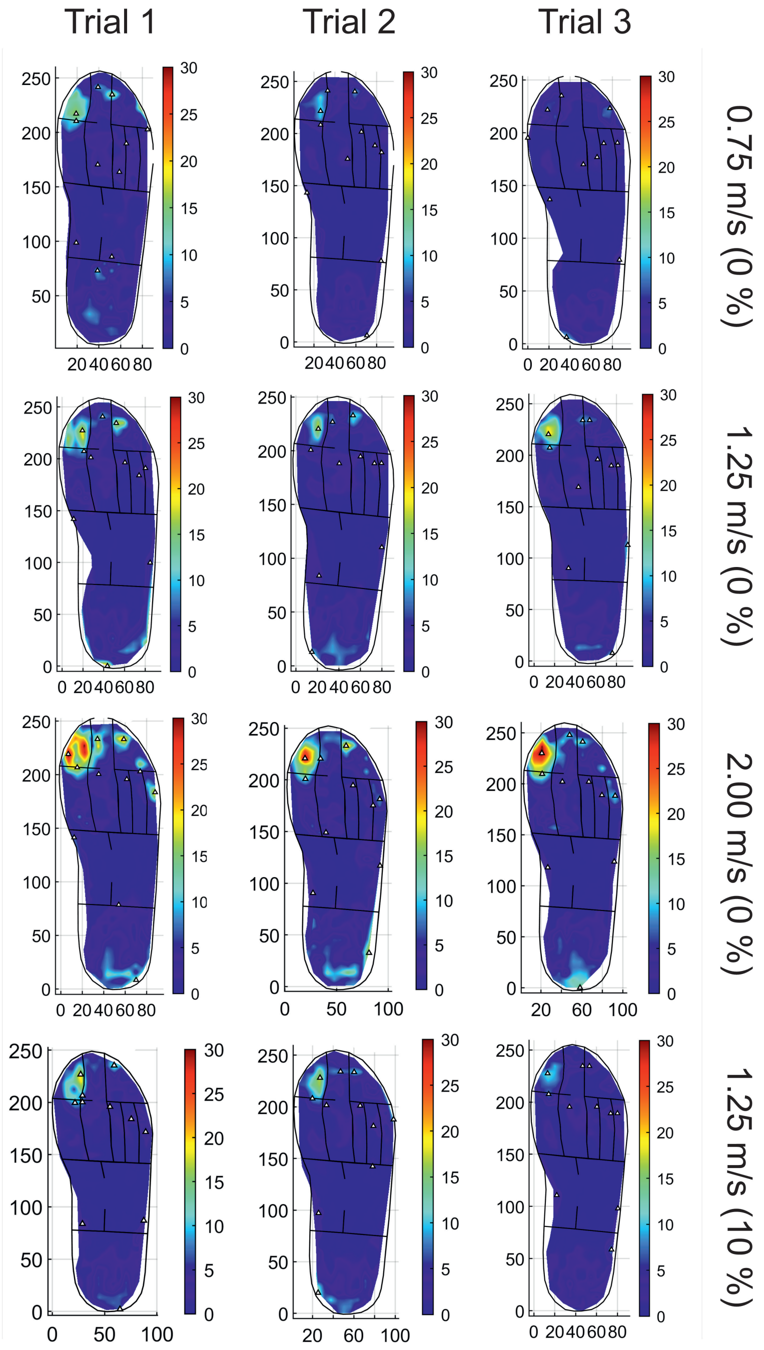

Figure S1. Strain profiles for repeated trialled speeds and inclinations for P01.

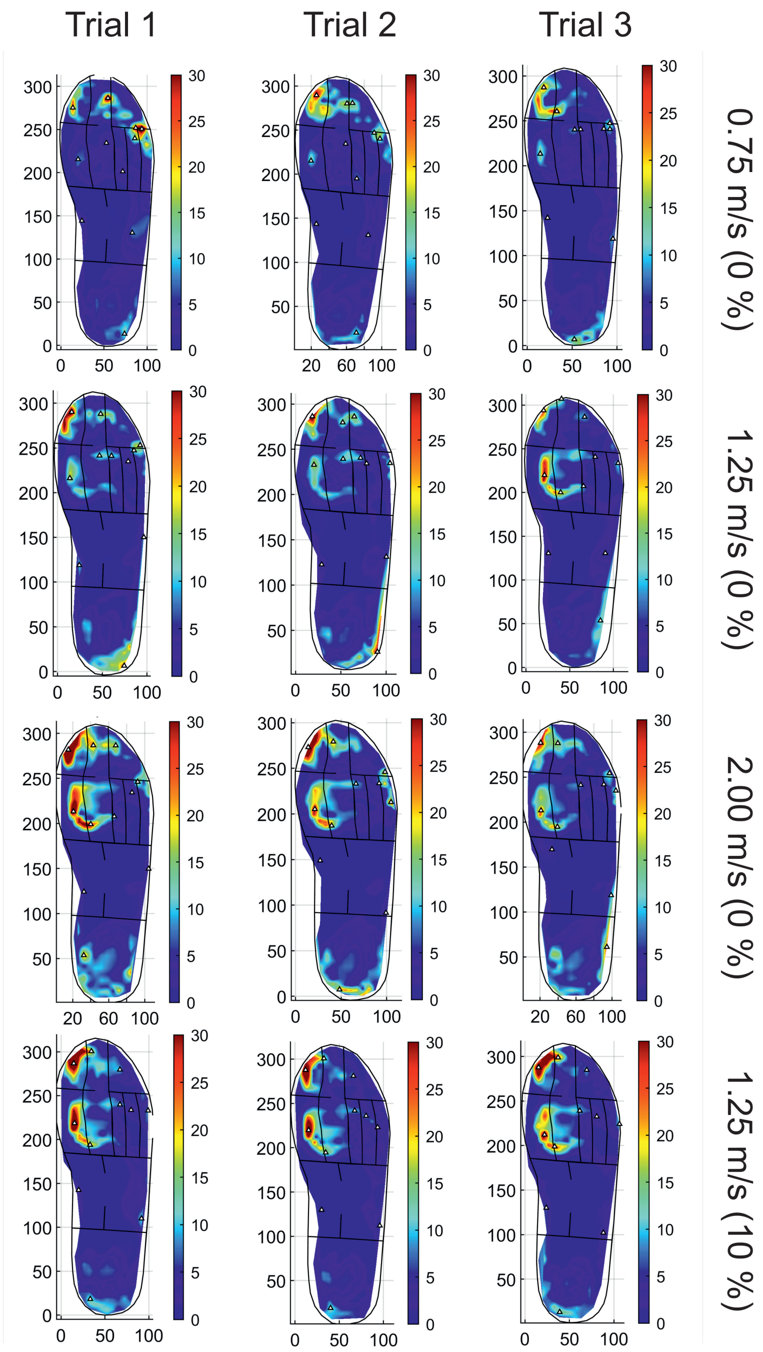

Figure S2. Strain profiles for repeated trialled speeds and inclinations for P02.

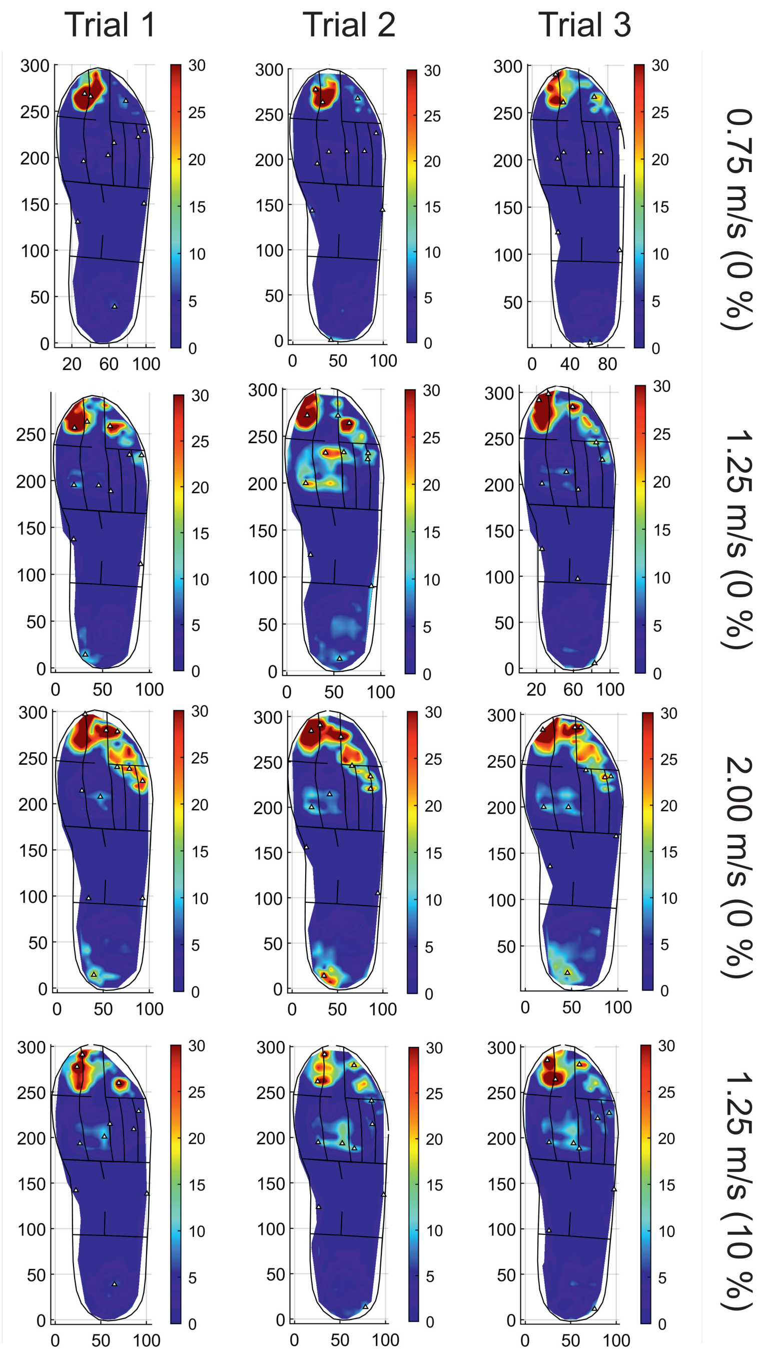

Figure S3. Strain profiles for repeated trialled speeds and inclinations for P04.

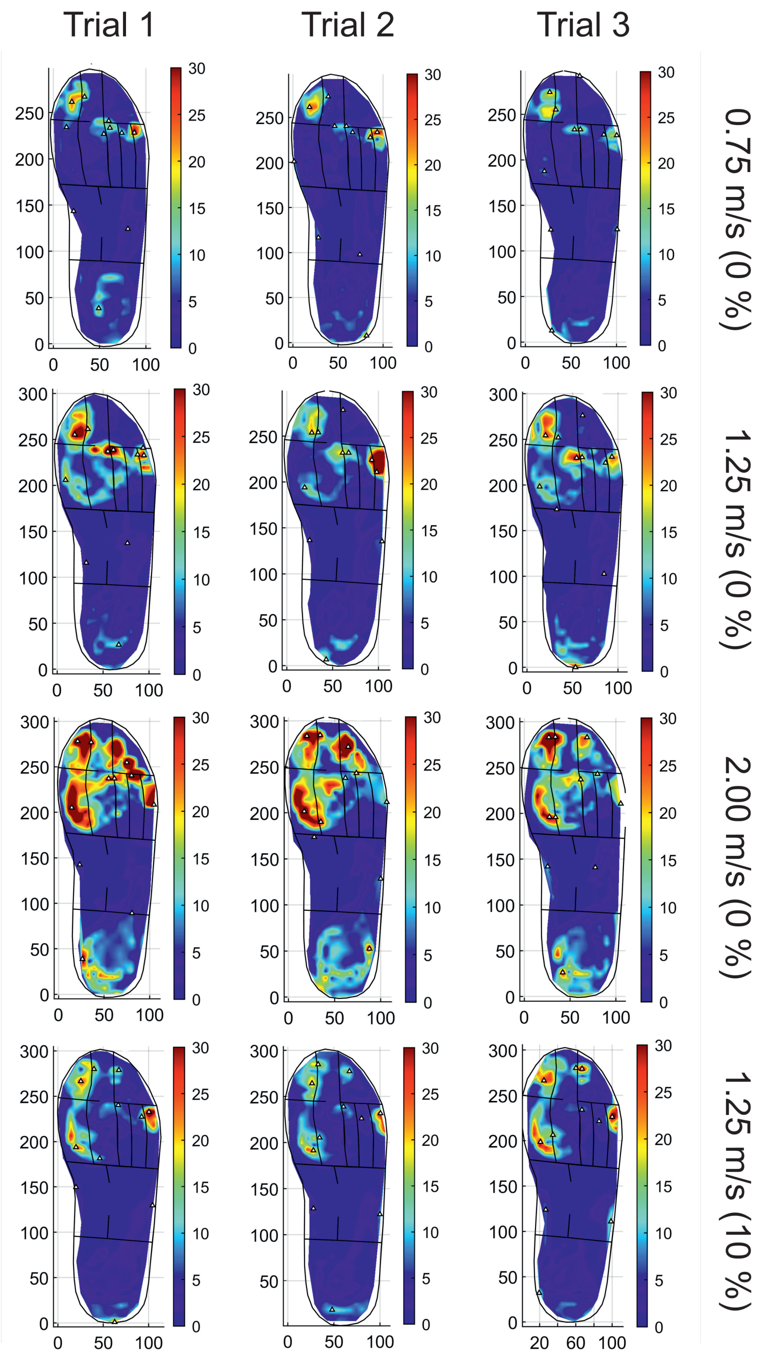

Figure S4. Strain profiles for repeated trialled speeds and inclinations for P05.
